# A Comparison of Four Molecular Methods for Detection of Aflatoxin-Producing *Aspergillus* in Peanut and Dried Shrimp Samples Collected from Local Markets around Pathum Thani Province, Thailand

**DOI:** 10.1155/2020/8580451

**Published:** 2020-12-24

**Authors:** Ratchanok Kumsiri, Panan Kanchanaphum

**Affiliations:** ^1^Pathobiology Unit, Faculty of Science, Rangsit University, Pathumthani, Thailand; ^2^Biochemistry Unit, Faculty of Science, Rangsit University, Pathumthani, Thailand

## Abstract

*Aspergillus flavus* is an aflatoxin-producing fungus which is poisonous to humans and animals when consumed. Detecting the fungus can help to prevent this danger. The four molecular methods, namely, conventional isothermal amplification (LAMP), PCR, quantitative LAMP (qLAMP), and qPCR, were compared to determine their efficiency for *A. flavus* detection. Thirty samples of peanut and dried shrimp were collected from 15 markets around Pathum Thani Province in Thailand. The samples were artificially infected with 10^8^ conidia/ml of *A. flavus* for 1 hr and enriched for one day to represent real contamination. The results show that the sensitivity detection for *A. flavus* in PCR, LAMP, qPCR, and qLAMP was 50 ng, 5 ng, 5 pg, and 5 pg, respectively. *Aspergillus* in 30 peanut and dried shrimp from the market was detected by all four methods. The detection rate was about 20%, 60%, 100%, and 100% with PCR, LAMP, qPCR, and qLAMP, respectively. The molecular detection technique, especially LAMP, qPCR, and qLAMP, can detect this pathogenic fungi very rapidly with high sensitivity and reliability in comparison to conventional PCR.

## 1. Introduction

Food safety problem is the important problem that is a threat to the health of the population [1, 2], especially in the developing countries. The major trouble of food safety is food poisoning by the contamination of microorganisms such as bacteria and fungi. This study emphasizes only the fungus, particularly genus *Aspergillus*.

The genus *Aspergillus* includes more than 600 species; about 40 species are known to cause disease in humans [3]. This dangerous species produces aflatoxins. Aflatoxins are a group of mycotoxins that display acute immune and hepatotoxicity in humans. Aflatoxins also exhibit very high carcinogenic potential following chronic exposure [4, 5]. *Aspergillus* section *Flavi* includes species capable of producing a wide array of toxins among which aflatoxins are the most important for food safety. Aflatoxigenic fungi have contaminated many kinds of food and cereal [6, 7] such as dried fruits, nuts, edible seeds [8], rice, spices [9], and herbs [4, 10]. Food and feed are particularly susceptible to colonization by aflatoxigenic *Aspergillus* in warm and dry weather. Aflatoxigenic fungi can produce toxin at several stages of the food chain, such as preharvest, processing, transportation, and storage [11, 12].

The aflatoxigenic fungi species is divided into three sections of the fungal genus *Aspergillus,* i.e., sections *Flavi, Nidulantes,* and *Ochraceorosei* [13]. Among these, section *Flavi* includes 16 species capable of producing a wide array of toxic compounds [14]. However, *A. flavus* and *A. parasiticus* are significant because they produce aflatoxin in food [15]. *A. flavus* and *A. parasiticus* have different toxicogenic profiles. *A. flavus* produces aflatoxin B1, B2, cyclopiazonic acid, aflatrem, 3-nitropropinic acid, sterigmatocystin, versicolorin *A*, and aspertoxin, whereas *A. parasiticus* produces aflatoxin B1, B2, G1, G2, and versicolorin *A* [16].

In this study, the *Aspergillus* norsolorinic acid reductase gene (NOR1) was selected for molecular detection with PCR, qPCR, LAMP, and qLAMP. The gene is involved in aflatoxin B synthesis. The nor1 encodes norsolorinic acid reductase. This enzyme converts norsolorinic acid to averantin. However, researchers have investigated other genes involved in the biosynthesis of aflatoxins. The multiplex PCR technique with three genes, *aflM*, *aflP,* and *aflR,* was used to detect *Aspergillus flavus* in peanut, corn, and three insect species commonly found in stored grains [14]. Reddy et al. [15] used *aflR* and *aflO* to detect *A. flavus* contamination in rice samples. However, not only genes were used in the aflatoxin biosynthesis to determine the *Aspergillus* contamination.

Therefore, efficient methods of identifying and detecting aflatoxigenic fungi in food and raw ingredients should be developed. Currently, molecular methods such as polymerase chain reaction (PCR), real-time PCR or quantitative PCR (qPCR), and loop-mediated isothermal amplification (LAMP) are effective for detecting food contamination by microorganisms.

Nowadays, LAMP has become the interesting method to replace PCR due to its more rapid, simple, and specificity reaction. In this present time, LAMP has been developed and used in many applications, such as for sex determination in human [17], detection of pork meat in Halal food [18], detection of *Yersinia enterocolitica* in pork meat [19], and detection of *Salmonella* infection in chicken samples [20] This method was performed under isothermal conditions in the temperature range 60°C–65°C for 60 minutes [21]. There are two sets of primer; inner primer and outer primer sets used in LAMP were specific at six different DNA sequences within the target DNA, and primary DNA amplification was begun by the inner primer set. The characteristic intermediary DNA structure formed by LAMP, called a stem-loop DNA fragment, was generated, and large amounts of DNA products were produced by an autocycle reaction [22].

Then, this research aims to compare the efficiency of the PCR, qPCR, LAMP, and qLAMP methods for detecting aflatoxin-producing species within *Aspergillus* section *Flavi* and to apply these methods to detecting fungi purified DNA, spore suspension artificially contaminated samples, and naturally contaminated peanut and dried shrimp collected from local markets around Pathum Thani province, Thailand.

## 2. Materials and Methods

### 2.1. Fungal Isolates and Culture Conditions

Assist. Prof. Dr. Ladawan Wasinpiyamongkol from the Microbiology Department, Faculty of Science, Rangsit University, Thailand, kindly supplied all the fungal cultures (*A. flavus, A. Niger, Pennicillium oxalicum, Trichoderma asperellum,* and *Rhizopus* sp.). The cultures were all maintained on potato dextrose agar (PDA, Difco, BBL/USA) supplemented with 50 mg/l of Choloramphenicol and 25 mg/l of Gentamycine. The agar plates were incubated at 28°C ± 2°C for 7–10 days and then monitored daily for the appearance of fungal colonies. The DNA was extracted from the isolated fungi using a fungal DNA extraction kit (PrestoTM Mini gDNA Yeast kit; Geneaid, New Taipei City, Taiwan), and the DNA concentration was measured using a NanoDrop 2000c Spectophotometer (Thermo Scientific).

### 2.2. Primer Design for PCR and LAMP

PCR primers set (Nor1-F and Nor1-R) and LAMP primers set (F3-nor1, B3-nor1, FIP-nor1, and BIP-nor1) were designed for specific to norsolorinic acid reductase gene (NOR1) (adapted from Ludwig Niessen and colleagues [4]). The nucleotide sequences of each primer are shown in Table 1.

### 2.3. PCR and qPCR Reactions

A PCR assay targeting the Norsolorinic acid reductase gene was performed in parallel with the LAMP primers to compare the detection efficiency, as shown in Table 1. SYBR green I dye was used to enhance the specificity in the qPCR reaction. The PCR amplification contained 1 × Taq DNA polymerase buffer, 1.2 mM dNTPs, 0.8 *μ*M F3 and B3 primers, 8 U Taq DNA polymerase (New England Biolabs), and 5 ng of each DNA extract as a template in a final volume of 25 *μ*l. The cycling conditions comprised of a single initial denaturation at 95°C for 3 min followed by 35 cycles at 90°C for 30 sec (denaturation), 56°C for 30 sec (annealing), 72°C for 30 sec (extension), and a final extension step at 72°C for 5 min. After the PCR amplification, the products of 550 bp were analyzed by electrophoresis using 1.5% agarose gel and were analyzed by Gel Doc^TM^ XR+ with Image Lab^TM^ Software (BIO RAD, USA).

### 2.4. LAMP and qLAMP Reaction

All LAMP reaction were performed followed the methods of Kanchanaphum and Vichaibun [20] containing 5 mM MgSO_4_, 400 mM betaine (Sigma), 1.2 mM dNTPs, 0.8 *μ*M F3 and B3 primers, 2 *μ*M FIP and BIP primers, and 8 U Bst DNA polymerase (New England Biolabs). After the LAMP reaction, the products were analyzed by electrophoresis using 1.5% agarose gel and were analyzed by Gel Doc^TM^ XR+ with Image Lab^TM^ Software (BIO RAD, USA.). The qLAMP amplification was performed by adding 0.5 *μ*L of SYBR green I dye (Invitrogen, Carlsbad, CA) to the normal LAMP reaction.

### 2.5. The Specificity and Sensitivity of the PCR, qPCR, LAMP, and qLAMP

For the specificity testing, 5 *μ*g/*μ*L DNA templates of *A. flavus* and non-*A. flavus* were subjected to PCR and LAMP methods.

The DNA sensitivity testing for *A. flavus* was 10-fold serial dilution from 5 *μ*g/*μ*L to 50 pg/*μ*L for all four methods.

### 2.6. Artificial Contamination of the Peanut and Dried Shrimp with *A. flavus*

For the experiment, the peanut and dried shrimp samples were transferred to a sterile container, and then, the methods of Kanchanaphum and Vichaibun [20], Shapira et al. [23], and Siruguri et al. [24] were followed. Then, all samples were dried in a laminar hood under ultraviolet light for 3 min. After that, the peanut and dried shrimp were inoculated with 10^8^ conidia/ml of *A. flavus*. The samples were incubated for 1, 3, 6, and 24 hrs., respectively. Then, the infected samples were cultured in PDA for 1 and 2 day for enrichment time. After that, fungi DNA extraction was performed.

### 2.7. The Detection of *A. flavus* in Peanut and Dried Shrimp Samples Gathered from Local Markets

Thirty peanut samples and thirty dried shrimp samples were collected from 15 local markets in Pathumthani Province, Thailand. After the purchase, all of the samples were stored in an ice box and immediately delivered to a laboratory for DNA extraction and further experiments.

## 3. Results

### 3.1. The Specificity Test of the PCR and LAMP

**Figure 1:**
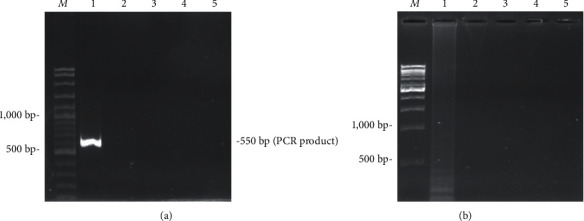
The specificity test of PCR (a) and LAMP (b). Lane *M* = DNA marker. Lane 1 = *Aspergillus flavus* DNA. Lane 2 = *Aspergillus Niger* DNA. Lane 3 = *Pennicillium oxalicum* DNA. Lane 4 = *Trichoderma asperellum* DNA. Lane 5 = *Rhizopus sp.* DNA.

The results of the specificity tests for the PCR and LAMP methods are shown in Figures 1(a) and 1(b), respectively. Both methods demonstrated 100% specificity when tested with non*-Aspergillus flavus* strains.

### 3.2. The Sensitivity Test of the PCR, qPCR, LAMP, and qLAMP

**Figure 2:**
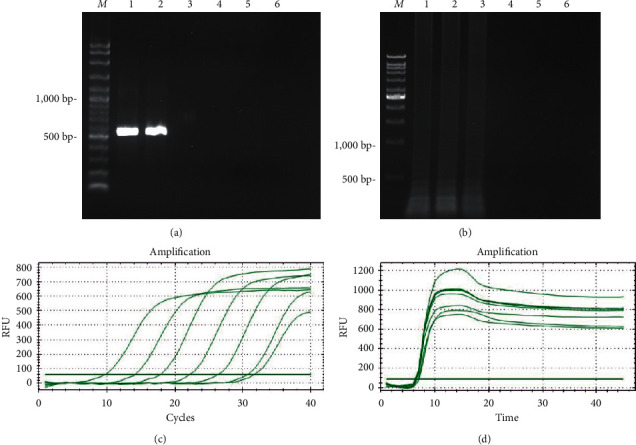
The sensitivity test of PCR (a), LAMP (b), qPCR (c), and qLAMP (d).

**Figure 3:**
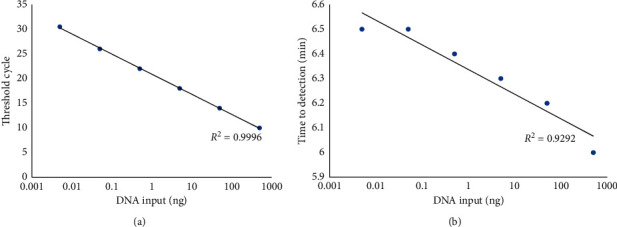
The standard curve of cycle threshold in qPCR (a) and time of detection in qLAMP (b).

Ten-fold dilutions of the DNA template were used to compare and determine the detection limits of all four methods. For the PCR assay, 550 bp PCR product was found in 1 and 2, as shown in Figure 2(a). The detection limit for the PCR assay was 50 ng. The detection limit for the LAMP reaction was 5 ng, as shown in Figure 2(b). Therefore, the sensitivity for both qPCR and qLAMP was 5 pg, as shown in Figure 2(c) and 2(d). The *R*^2^ value from standard curve of cycle threshold from qPCR was 0.9996, and the *R*^2^ value from standard curve of time to detection from qLAMP was 0.9292, as shown in Figure 3.

In Figure 2(a) and 2(b),

- Lane *M* = DNA marker
- Lane 1 = *Aspergillus flavus* DNA 5 *μ*g
- Lane 2 = *Aspergillus flavus* DNA 500 ng
- Lane 3 = *Aspergillus flavus* DNA 50 ng
- Lane 4 = *Aspergillus flavus* DNA 5 ng
- Lane 5 = *Aspergillus flavus* DNA 500 pg
- Lane 6 = *Aspergillus flavus* DNA 50 pg

So, the sensitivity of each method (PCR, qPCR, LAMP, and qLAMP) was summarized in Table 2.

### 3.3. The Detection of *A. flavus* in Spiked and Naturally Contaminated Peanut and Dried Shrimp Samples from the Markets

For artificial infection, the samples were incubated for 1 hr and then cultured in PDA for 1 day. The samples were incubated for 1, 3, 6, and 24 hrs., respectively. Then, the infected samples were cultured in PDA for 1 and 2 days for enrichment time. After that, fungi DNA extraction was performed.

A total of 30 peanut samples and 30 dried shrimp samples collected from 15 markets around Pathum Thani province, Thailand, were enriched for one day. After DNA extraction and amplification with all four assays, the results were obtained, as shown in Table 3.

## 4. Discussion

In this study, the *Aspergillus* norsolorinic acid reductase gene (NOR1) is selected for molecular detection with PCR, qPCR, LAMP, and qLAMP because it is highly effective at detecting the *A. flavus* contamination in food. These primers showed high specificity and sensitivity. Beyond the NOR1, the internal transcribed spacer regions 1 and 2 (ITS1 and ITS2) were also used to detect the Aspergillus. Levin [25] reported that the sensitivity of ITS1 and ITS2 PCR primer for *A. parasiticus* CBS 126.62 was 9.03 pg, while our result for the PCR detection using the nor1 gene target was 500 ng. Our sensitivity was quite low compared with Levin and colleagues [25]. So, the PCR condition such as the concentration of magnesium ion may be adjusted for the better sensitivity. The sensitivity of PCR primarily depends on three factors: the physicochemical condition of the reaction, the concentration of DNA template, and the specific primer [26]. However, our results for the LAMP reaction was 50 ng, which was quite similar to the findings of Luo et al. who used acl1 (ATP citrate lyase subunit 1) to determine the contamination [27]. Their sensitivity result for the LAMP reaction was 20 ng [27], which implies that the sensitivity of the LAMP method is better than the PCR method.

qPCR and qLAMP have been used to enhance performance. Freckle et al. [28] used the qPCR method to detect and characterized the *Aspergillus* species. Schabenreiter-Gurtner et al. [16] also used qPCR to detect the different *Aspergillus* and *Candida* species by targeting the ITS-2 region of the fungi. Both Frickle S. et al. [5] and Schabenreiter-Gurtner et al. [16] used hybridization probes to perform the qPCR detection, which was expensive and complicated.

Therefore, in this research, a method using SYBR green fluorescence was developed. This was more cost effective and less complicated than the hybridization probe method or the TaqMan method, which is based on dual labeled olignonucleotide. The results of this research demonstrated that the qPCR (50 pg) sensitivity detection was 10,000 times better than conventional PCR (500 ng) sensitivity detection. Like the qPCR method, the qLAMP method improved the efficiency of the conventional LAMP method. The performance of the qLAMP method (50 pg. of DNA) was 1,000 times better than the conventional LAMP method (50 ng. of DNA). These results were according to Marmiroli and Maestro [28]. They reported that the limit of detection by ethidium bromide staining is about 5 ng DNA (visual inspection) and 60 pg DNA by SYBR Green I [29]. The limit of detection by ethidium bromide staining is about 5 ng DNA (visual inspection) and 60 pg DNA by SYBR Green I [29].

From this study, it can be seen that the detection rate of *Aspergillus* infection in peanut and dried shrimp from the market were very low using PCR, 20% positive in peanut and 13.33% positive in dried shrimp (from Table 3). The main reason of high numbers of negative result is the limitation of PCR. The efficiency of PCR technique is 50 ng DNA of pathogen, while the efficiency of LAMP and both qPCR and qLAMP are 10 and 10^4^ times, respectively, higher than that of PCR. So, the LAMP has about 3 times proficiency than PCR, while qPCR and qLAMP have about 5–7.5 times proficiency than normal PCR (from Table 3).

The analysis' capability to detect low amount of *A. flavus* in peanut and dried shrimp was confirmed. On the other hand, all four methods required the complex equipment, such as a gel-electrophoresis apparatus or real-time PCR machine, to detect the DNA amplification. Further research could develop the lateral flow dipstick or LFD method to detect the DNA amplification product because it is easier and less complicated. The LFD method can also detect the amplification product without a gel-electrophoresis apparatus, which is useful for field studies.

## 5. Conclusions

The results show that PCR, LAMP, qLAMP, and qPCR methods could detect *Aspergillus* contamination in peanut and dried shrimp. Especially, both LAMP and qLAMP methods were more rapid, reliable, and robust for *Aspergillus* detection in the samples and may be useful molecular tools for routine testing. In addition, qLAMP and qPCR methods were the most efficient in terms of sensitivity.

## Figures and Tables

**Table 1:** List of primers for PCR and LAMP reactions.

Primer name	Sequence 5′-3′
F3-nor1	ACT GCG ACT CGG AAA GCG A
B3-nor1	GGA CTG CTG CAG CAT CAG
FIP-nor1	GGC CCA AAG TTC TGC GCC AT-C CAG ACA TTG CGG GAG GA
BIP-nor1	ACC ATG CCC CTC GAG GAT CT-G CGG GTT GCC TGA AAC AG
Nor1-*F*	CTTTTCTCCAACGTCCCAAA
Nor1-*R*	ACAAGAACCCTCGGACTGTG

**Table 2:** Sensitivity of the four detection methods to detect *Aspergillus* infection.

DNA concentration	5 *μ*g	500 ng	50 ng	5 ng	500 pg	50 pg
Method	—	PCR	LAMP	—	—	QPCR qLAMP

**Table 3:** The number of *Aspergillus* infected samples from 15 markets (30 samples) by the PCR, qPCR, LAMP, and qLAMP method.

Method	PCR	qPCR	LAMP	qLAMP
Positive samples (peanut)	6/30	30/30	16/30	30/30
Positive samples (shrimp)	4/30	30/30	18/30	30/30

## Data Availability

The data used to support the findings of this study are available from the corresponding author upon request.
